# The global landscape of country-level health technology assessment processes: A survey among 104 countries

**DOI:** 10.1016/j.hpopen.2025.100138

**Published:** 2025-03-27

**Authors:** Andrew J. Mirelman, Kratu Goel, Tessa Tan-Torres Edejer

**Affiliations:** Department of Health Financing and Economics, World Health Organization, Geneva, Switzerland

**Keywords:** Health technology assessment, Priority-setting, Institutionalization

## Abstract

To make progress towards universal health coverage (UHC), countries need to be able to develop and implement evidence-informed and inclusive processes for decision-making to inform what services are provided and purchased. Health Technology Assessment (HTA) processes are an established mechanism to support evidence-informed decision-making in the health sector. To assess the practice of HTA in countries, WHO conducted a global survey that explores the status of HTA. The survey is the largest source of information in terms of number of countries for describing global HTA status. The final sample analysed in this paper is the 104 countries (82% or 104/127) that responded “yes” to the question of having a systematic, formal health decision-making process at the national level. The results show that while many countries have HTA bodies in place, these serve different functions. While there are more established processes in higher income country groups, even those show room for improvement in areas such as social participation and appeals. Lack of awareness of the importance of HTA and institutionalization were the top two barriers to HTA utilization. There is further progress that needs to be made in HTA globally. Countries need to build on available guidance to ensure the appropriate elements are in place for sustainable country decision-making processes and develop more understanding of how they vary across settings.

## Highlights

• Health technology assessment (HTA) processes are being developed around the globe.
• This survey contains information on HTA processes in 104 countries.
• The results provide guidance for planning further efforts for HTA institutionalization.

## 1 Background

To make progress towards universal health coverage (UHC), countries need to be able to develop and implement evidence-informed, legitimate, and inclusive processes for decision-making that can inform what services are provided and purchased, and at what price. Health decision-makers face tough choices when deciding amongst the numerous available interventions to cover to meet the health needs of their populations and balance multiple health system objectives such as fairness and efficiency. Each decision also has an implication on what can be covered elsewhere in the system; that is, it has an opportunity cost.

To support health decision-making, Health Technology Assessment (HTA) processes have existed for several decades and as an established mechanism for evidence-informed priority setting in the health sector, and it has even been included in multiple WHO resolutions such as WHA 67.23 in 2014 [1]. HTA is defined as, “a multidisciplinary process that uses explicit methods to determine the value of a health technology at different points in its lifecycle. The purpose is to inform decision-making in order to promote an equitable, efficient, and high-quality health system.” [2]. The term “health technology” is defined broadly as any health service or intervention. HTA can be applied to many decision-making scenarios such as whether to include a new medicine into a health coverage scheme, rolling-out broad public health programs (such as immunization or cancer screening), informing pricing for medicines, and formulating clinical guidelines [3].

Despite commonalities, HTA processes vary significantly from country to country. There have been several efforts to understand the status of HTA in countries around the world, including information on the institutional arrangement, processes, and barriers. In Asia, there have been studies to understand the landscape of HTA systems and practices, which find that processes are implemented differently and that there is often a lack of adequate resources [4], [5]. In Europe, the variation in practice and barriers to production and use of HTA has been explored in more depth through the EUnetHTA project and in groups of countries such as Central, Eastern, and South-Eastern Europe (CESEE) countries, non-European Union (EU) countries, and the Balkan countries [6], [7], [8], [9]. For Latin America and the Caribbean region, a survey of 30 countries found that despite much development in the area of HTA, the uptake of formal processes in the region remained relatively low [10]. In the Middle East region, HTA is still relatively new, and a survey of countries showed that there is demand for further institutionalization [11]. In a survey across low- and middle-income countries, a landscape assessment of HTA concluded that while HTA practices were growing in low- and middle-income countries, there are critical gaps in incorporating the recommendations from HTA into policy [12]. Finally, in a survey of 27 established HTA agencies around the world, it was found that while there was relatively well-established guidance for assessment, other areas of governance, appraisal and monitoring lacked definitive guidance [13].

HTA development around the world requires that processes are institutionalized in a manner that is robust and sustainable. A WHO framework on how to institutionalize HTA features a five-part framework that includes: 1) establishing a mandate, 2) establishing a legal framework, 3) establishing institutional arrangements, 4) procedural aspects of assessment and appraisal, and 5) monitoring and evaluation of the HTA mechanism [3]. To assess the practice of HTA in countries, the WHO conducted a global survey in 2020 and 2021 that builds on this framework and explores the status of HTA. To our knowledge, the survey is the largest source of information in terms of number of countries included for describing the global status of HTA. Using the institutionalization framework, the objective of this paper is to show the results from the survey in order to understand the current landscape of HTA practice.

## 2 Methods

The questionnaire was updated from a previous survey and sections included: Institutions and Governance, Available Resources, Assessment, Appraisal, Recommendation and Barriers [14]. The questionnaire was also refined based on consultations with experts at WHO headquarters, as well as with regional and country offices and through piloting with respondents in 10 countries (see annex).

To obtain responses for a given country, a systematic approach was used to identify a principal respondent. Firstly, respondents were identified and contacted from an officially issued circular letter based on the 2014 WHA resolution 67.23. If respondents indicated that they were not available, or did not respond to multiple contact attempts, a protocol was followed with WHO regional and country offices to identify experts that could answer on behalf of the country's situation. When no respondent was identified by these means, national experts were identified from professional networks to facilitate a connection with potential respondents.

The survey was issued electronically via WHO’s Dataform software that is based on the LimeSurvey platform. Each respondent was emailed a link to access the survey along with supporting documentation. Respondents were also informed about the process to coordinate a response from multiple people, if required. After submission, each respondent was sent a copy of their answers for confirmation. Further validation occurred by reviewing responses for answers that appeared inconsistent and following up with respondents. The only deviation to the approach for respondent identification and implementation was in the WHO region of the Americas where an internal survey with significant overlap was being conducted by the Pan-American Health Organization (PAHO). The PAHO survey had an objective to evaluate the capacities in countries in that region for evidence-based policies related to medicines and health technologies. In this case, responses from countries that previously responded to the PAHO survey were copied over by an expert into WHO survey’s platform for questions that were identical.

To facilitate responses, the survey was made available in 5 UN Languages: English, French, Spanish, Arabic, and Russian. The translations were conducted by professional UN translators and technical terminology was reviewed during the pilot testing phase.

The survey data was cleaned and analysed descriptively according to the five sections of the institutionalization framework. Further details on data cleaning and translation have been provided in Annex 1. Within the sections, some questions were also broken down according to World Bank country income status including high-income countries (HIC), upper-middle income countries (UMIC), lower-middle income countries (LMIC) and low-income countries (LIC), in order to draw conclusions according to country resource levels [15]. For several questions, results are also provided by different intervention categories that include: diagnostic tests, medical device, medical procedure, medicines, and population-level health interventions.

## 3 Results

### 3.1 Response rates, sample selection, and decision functions

Accounting for the specific sampling arrangement with PAHO countries described above, there were surveys sent to 144 countries and a resulting 104 responses, giving a 72 % response rate. Adding the 23 countries from PAHO gives 127 responses received overall. Table 1 shows the breakdown of responses for these by country income status. The final sample analysed in this paper is the 104 countries (82 % or 104/127) that responded “yes” to the question of having a systematic, formal health decision-making process at the national level regardless of whether they are called “HTA” or not. There were 62 % (64/104) of countries that said that they call this process HTA. For the remainder of the paper, we use the general term “HTA” to refer to all 104 country responses. When countries responded “yes” to having both a national and sub-national process, they were asked to respond just for the national process. Two countries in our sample, Costa Rica, and Maldives, responded to only having sub-national processes, however on further clarification with the respondents, these countries were added to the final sample as their processes were in fact national in scope.

**Table 1:** Survey responses and presence of systematic, formal process by income status.

**Country Income Status**	**Surveys Submitted**	**Existence of systematic, formal process to support health decision-making at the national level? (n = 127)**		**Do you refer to this process as Health Technology Assessment (HTA)? (n = 104)**		
		Yes	No	Yes	No	N/R
HIC	44	36	8	28	5	3
UMIC	29	25	4	18	5	2
LMIC	37	30	7	13	17	0
LIC	17	13	4	5	7	1
**Total**	**127**	**104**	**23**	**64**	**34**	**6**

The results for many of the questions are seen in Table 2. An additional table which reports all of the results by country income group is also found in the annex.

**Table 2:** Overall results to selected survey questions.^a^^,^^b^

**Variable**	**Response option (when applicable)**	**Yes**	**No**	**N/R**
**Mandate and Legal Framework**				
**Functions (n = 104)**	**Planning and budgeting**	81	19	4
	**Clinical practice guidelines**	78	21	5
	**Design of Health Benefit Packages**	66	34	4
	**Protocols for public health programmes**	59	38	7
	**Public Procurement of Medicines**	56	45	3
	**Indicators of quality of care**	56	43	5
	**Pricing/pricing negotiations**	54	48	2
	**Objectives for P4P schemes**	31	66	7
	**Other**	19	0	85
**Legislative requirement (n = 104)**		54	34	16^a^
**Binding results (n = 104)**		32	17	55^a^
**Institutional Arrangements**				
**Collaboration within the country (n = 67)**	**Ministries or other Government Institutions**	54	11	2
	**Academia/University**	46	19	2
	**Professional associations**	37	29	1
	**Hospital**	31	32	4
	**Patient Associations**	21	42	4
	**Industry**	17	44	6
	**Other**	19	42	6
**Collaboration outside the country (n = 53)**	**Ministries or other Government Institutions**	28	22	3
	**Academia/University**	18	30	5
	**Professional associations**	8	39	6
	**Hospital**	3	44	6
	**Industry**	2	45	6
	**Patient Associations**	1	46	6
	**Other**	27	24	2
**Standard Methodology/Process Guideline (n = 104)**		52	39	13
**Mechanism for translation (n = 104)**		41	37	26
**Provision for rapid assessment − Non-Emergency (n = 104)**		50	32	22
**Provision for rapid assessment – Emergency (n = 104)**		52	28	24
**Allocated public-sector budget (n = 104)**		59	33	12^a^
**Private Funding (n = 104)**		15	62	27
**Procedural aspects of Assessment, Appraisal and Recommendation**				
**Economic evaluation guidelines (n = 104)**		41	43	20
**Officially endorsed Cost-Effectiveness Threshold (n = 104)**		19	63	22
**Variation in threshold (n = 19)**		8	10	1
**Stakeholders have equal voice in Appraisal (n = 104)**		55	14	35
**Members of Appraisal body provide a conflict of interest (n = 104)**		60	16	28
**Non-represented stakeholders allowed to react (n = 104)**		42	30	32
**Separate entity for Recommendation (n = 104)**		48	56	0
**Possibility of Appeal (n = 104)**		22	11	71
**Publication of Recommendations (n = 104)**	**Minutes of the meetings**	29	68	7
	**Assessment reports**	48	53	3
	**Recommendations (or decisions where relevant)**	48	54	2
	**Rationale for the decision**	27	72	5
	**Others**	12	82	10
	**No outputs from the recommendation process are published**	16	77	11
**Monitoring and Evaluation**				
**Indicators to assess impact (n = 104)**		32	45	27
**Criteria to assess impact (n = 32)**	**Health outcomes**	24	6	2
	**Variation in practice (before/after)**	22	8	2
	**Cost of medical practice**	19	12	1
	**Changes in health from patient view**	19	10	3
	**Variation in practice (current/recommended)**	18	11	3
	**Level of technology diffusion**	16	14	2
	**Changes within organizations or facilities**	14	15	3
	**Changes in the law**	10	19	3
	**Other**	3	0	29

a For these variables, the N/R values also included responses to the option “I don’t know”. These were merged into the N/R column for visual convenience with respect to this table, and these values never exceeded 5% of the total sample of observations (i.e. more than a total of 5 respondents).

b Complete results of the survey alongside country profiles have been published on WHO’s website at the following link − https://www.who.int/teams/health-financing-and-economics/economic-analysis/health-technology-assessment-and-benefit-package-design/survey-homepage.

### 3.2 Mandate and legal framework

Two foundational elements for institutionalizing HTA processes involve establishing a mandate and a legal framework. To understand their mandates, the survey asked countries for which functions HTA processes are being used. The top three functions included: planning and budgeting (78 %, 81/104), clinical practice guidelines (75 %, 78/104), and design of health benefit packages (65 %, 66/104). Further functions include protocols for public health programs, public procurement of medicines, quality of care indicators and pricing of medical technologies, which were reported by around 50 % − 60 % of countries for each response. The least popular response, reported by 30 % (31/104) of the sample was for determining the objectives of pay-for-performance schemes.

For legal provisions, overall, 54 (52 %) countries said that there was a legislative requirement to consider the results of a decision-making process in coverage and health benefit package decisions. By income group, high-income countries and upper-middle-income countries were more likely to have legislative and/or regulatory requirements (67 % and 60 % of these countries, respectively), while lower-middle-income and low-income countries had lower rates (30 % and 46 %, respectively). Overall, fewer countries, 32 (31 %), mentioned that the results of the decision-making process are considered binding by law. By percentage, this was found to be more prevalent in HICs, although it was relatively low across all income groups and had a high non-response rate.

### 3.3 Institutional arrangements

#### 3.3.1 Available resources: staffing, collaborations and budgets

To understand staff resources, the survey asked how many staff are employed by the HTA entity. A small number of countries, 3 (3 %), reported having less than one full-time equivalent (FTE) and 20 (19 %) countries reporting having 1–5 FTE. The largest number of countries, 28 (27 %), reported having 6–20 (FTE). For the other options, 8 (8 %) and 6 (6 %) countries reported having 21–50 and 51–100 FTEs respectively, while 12 (12 %) countries reported having over 100 FTEs and 26 (25 %) countries did not respond.

Many HTA organizations develop and maintain formal collaborations with partners that are internal and external to their country. These collaborations can augment the ability to conduct assessments by facilitating knowledge sharing, transfer of resources (both human and financial), capacity building, and other functions. Although it is difficult to say by the number of collaborations whether an HTA mechanism has achieved more or less institutionalization, 73 (71 %) countries reported having at least some type of collaboration while 47 (45 %) countries mentioned collaborating with partners both internally and externally. Twenty (20 %) countries mentioned collaborating exclusively with internal partners, and 6 (6 %) countries said they only collaborated with external partners. A further 12 (12 %) responded that they do not collaborate with any partners at all, while 19 countries provided no response to this question.

An allocated public sector budget for the production of HTA assessments can provide more stable and predictable funding. It is also a sign that systems are more institutionalized. In response to a question on whether there is an allocated budget from the public sector for their HTA or decision-making process, 59 (57 %) responded in the affirmative and 31 (32 %) countries responded that they did not have any budget from the public sector. A further 15 (14 %) countries reported having private sources of funding for their overall budget for HTA bodies, with the most common source of the private funding reported as donors in 8 of those 15 countries. By income group, it is seen that more HICs, 25 (74 %) have an allocated public sector budget, while UMICs and LMICs have this in fewer countries (13 or 52 % in UMIC and 17 or 57 % in LMICs), and only 4 (31 %) of LICs.

#### 3.3.2 Roles and professional backgrounds

Another element of institutional arrangements is understanding which stakeholders participate in the process at various stages. For nominating interventions, most countries 67 (64 %) said that this was done by the Ministry/Department of Health or National Health Service, with the second most common choice being an HTA committee or entity with 41 (39 %) countries. Fewer countries said that either patient organizations 11 (11 %), civil society 9 (9 %) or Industry/Pharma 6 (6 %) were responsible for nominating interventions.

**Fig. 1:**
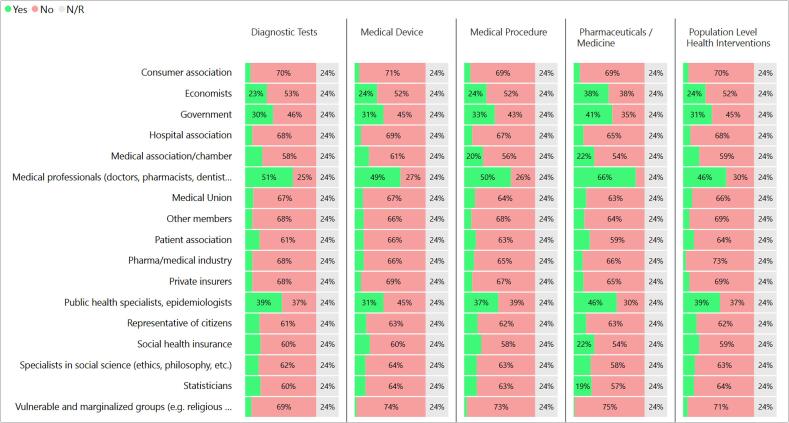
Professional backgrounds and representation of stakeholders involved in the appraisal of applications by intervention category. (n = 104).

Another element of understanding different stakeholder roles is to understand who is involved in the appraisal step of the process. Fig. 1 shows results for the stakeholder backgrounds and representation of those conducting the appraisal across different intervention categories. Overall, medical professionals are the most frequently included, followed by public health specialists, government officials and economists. The results are consistent across all intervention categories. The survey asked about a broad range of stakeholders outside of these four groups, and the results show that they are not included to a high degree in any of the processes for which responses were received.

#### 3.3.3 Translation and rapid decision-making

Translating results from one setting and making them applicable to another is also possible within HTA processes. It is also a way to address the need to produce evidence for tight policy windows without having to invest in expensive data collection or de novo analysis [16]. However, it should also be used with care, and the costs and benefits of whether to undertake analysis, or conduct a translation need to be assessed. Countries also reported on whether they had mechanisms for translating or contextualizing evidence from other settings. While more countries 41 (39 %) reported having such a mechanism than those that said they did not 37 (36 %), this difference was quite small. By country income group, there was not much difference in the response rate of countries. LICs had the highest percentage that said they had such a mechanism 6 (46 %) though the limited sample size in this income group should be noted.

A further element of institutional arrangements is to assess how these processes function outside of their “standard” model, that is, when decision-making may need to be conducted more rapidly. Fifty (48 %) countries reported having a provision for rapid assessment and decision-making in non-emergency contexts, and 32 (31 %) countries reported that they did not have such a process. Meanwhile, 52 (50 %) countries further mentioned that rapid assessments and decision-making were available in the case of a disaster or emergency, such as a pandemic. There were 28 (27 %) countries that responded that they did not have a rapid process for disasters or emergencies.

### 3.4 Procedural aspects of assessment, appraisal, & recommendation

The procedural aspects of HTA processes are typically broken down into three main steps: assessment, appraisal, and recommendation. Assessment includes generating and synthesizing evidence for given interventions while appraisal involves dialogue, generally in the form of deliberation, among multiple stakeholders to prioritize and come to a consensus. The recommendation step is then taking the result of the appraisal process forward for decision-making. Understanding the characteristics of these procedures and how they are conducted in different settings can uncover areas for improvement and learning across countries.

In terms of how much time assessments take to conduct, 67 countries responded overall. The most common answer was 3–6 months with 22 (33 %) countries, followed by 6–12 months with 19 (28 %) countries and 1–3 months with 18 (27 %) countries. The remaining 8 (12 %) countries said that assessments usually take more than a year. While having an overall sense of the time it takes to conduct HTA assessments is helpful, it should be noted that the time taken to conduct an assessment is not necessarily an indication of a higher quality product. There may be multiple types of HTA, such as full assessments and rapid assessments, that have different timelines, and which need to be fit-for-purpose for the HTA topics where they are applied.

**Fig. 2:**
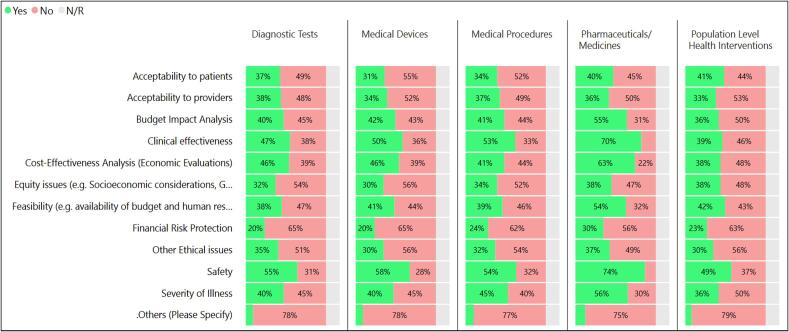
Criteria covered in the appraisal process for different intervention categories (n = 104).

The assessment and appraisal processes involve critical evaluation of interventions to relevant comparisons according to selected criteria. The survey attempted to understand which criteria were being used in appraisals and asked respondents to indicate among a list of 11 options of criteria that are potentially included. Fig. 2 shows that safety was the most common criteria overall, especially for pharmaceuticals and medicines. Clinical effectiveness, cost-effectiveness, and feasibility also rank as commonly included criteria, while the criteria of budget impact and severity were less likely to be included, though still prominent in some countries. The criteria of acceptability, equity, and financial risk protection were reported as being less frequently included. Future work should evaluate where certain criteria are included and excluded in the overall process, as for instance, some criteria such as budget impact and acceptability may feasibly enter farther downstream when decisions are considered at sub-national levels.

The criteria of cost-effectiveness can also be implemented in the assessment and appraisal through guidelines that detail methods or provide a reference case. Overall, a quarter of countries reported having such guidelines (24 %), although it is also worth noting that 50 % of countries did not respond to this question. There was also a clear pattern of responses seen by country income groups with HICs having the highest percentage of countries saying that they had such guidelines (41 %) followed by UMICs (28 %) and LMICs and LICs having a lower percentage of countries with economic evaluation guidelines (11 % and 10 %) respectively.

Globally, the use of cost-effectiveness thresholds for economic evaluation has been an area of substantial interest and ongoing research [17]. The survey posed several questions regarding cost-effectiveness thresholds. Only a few countries, 19 (19 %), said yes to using an officially endorsed cost-effectiveness threshold in their HTA or decision-making process. Ten of the nineteen countries with thresholds were from HICs, while there were 5 UMIC countries, 3 LMIC countries and 1 LIC country that used a threshold. Of all countries with a threshold, 8 (42 % of those that had a threshold) said that this threshold varies across different categories such as patients, diseases, or interventions. Of note, 17 (90 % of those that had a threshold) countries that reported using cost-effectiveness thresholds also reported having guidelines for conducting economic evaluation, which shows the link between having a documented processes with the administration of a cost-effectiveness threshold.

Accounting for conflict of interest and managing the voice and participation of different stakeholders are other important aspects of the appraisal process. Countries responded on whether members of the appraisal body were required to provide a declaration of conflict of interest. Here, 60 countries (58 %) said that these were required while 16 countries (15 %) said that they were not. In terms of stakeholder voice, 55 (53 %) countries said that there was equal voice for all stakeholders and 14 (13 %) said that there was not. Across all income groups, there was a higher percentage of countries that responded yes than countries that responded no. Countries were also asked whether stakeholders not represented in the process were allowed to formally react or comment. Here, 42 (40 %) of countries said yes versus 30 (29 %) saying no.

After the appraisal, and once a recommendation has been made, a further element to consider is appeal. There were 22 (21 %) countries that mentioned that there is a possibility to appeal the decision made by the recommendation body. Of these, 10 countries (45 % of those with appeal) said that this can be appealed before the decision-making body itself, and 8 countries (36 % of those with appeal) said that this can be appealed before a judicial court.

In order to gain insights about whether an HTA process is transparent and inclusive, it is important to know whether, and how, organizations share information. Countries answered that the most likely materials to be published and made publicly available were assessment reports and recommendations, with 48 (46 %) countries saying that these were provided. Meeting minutes and documentation of the rationale were mentioned by 29 (28 %) countries and 27 (26 %) countries respectively as being published and made available while 12 (12 %) countries reported publishing other types of materials. Finally, 16 (15 %) countries said that no outputs from the recommendation were published.

### 3.5 Monitoring and evaluation

Another aspect of the HTA process is whether monitoring and evaluation occurs, and the survey posed questions on whether the impacts of processes are assessed, and which indicators were used to assess impact. Forty-five (43 %) countries reported not including any indicators to assess the impact of their decision-making body while 32 (31 %) said that they do. Of those countries that assess their impact, the most frequent indicator included was “*Change in health outcomes over time (clinical changes)*” by 24 (75 %) countries, followed by “*Variation in practice before and after recommendation”* assessed by 19 (59 %) countries. More countries need to develop their monitoring and evaluation processes. This same result has been seen in other studies as well [13].

### 3.6 Barriers

Having further information on the barriers to HTA is necessary for planning and targeting activities for further development. The survey asked about barriers to the use of and production of HTA with results shown in a Figure in the supplementary materials. For barriers to use, the top-ranked barrier was ‘awareness of the importance of HTA’, mentioned by 38 (38 %) countries. The second and third most commonly top-ranked barriers in this category were ‘institutionalization of HTA’, reported by 17 (17 %) countries, and ‘political support’, reported by 11 (11 %). By income group, the top reported barrier was awareness of the importance of HTA in LICs, UMICs and HICs, while institutionalization was the top ranked barrier for LMICs. Institutionalization and the lack of qualified human resources were also frequently reported as the second- and third-ranked barrier among all countries. Further results were also reported about barriers to HTA production and training needs. These are reported in the annex.

## 4 Discussion

Throughout the last few decades, HTA has been spreading around the globe, while at the same time, there is much progress to be made. HTA entities and processes are not just the domain of high-income countries in Europe and North America. Many low- and middle-income countries have embarked on a path towards developing HTA processes; however, it should be acknowledged that HTA is a complex process with high upfront funding commitments and benefits can be uncertain or difficult to quantify [18]. The challenges for HTA in low- and middle-income countries may also be different than those for HTA in HICs, which can explain why models for implementation of HTA differ [19]. In another broad survey of 50 established HTA agencies, it was found that elements such as the key players, time to implementation, the top-down/bottom-up nature, and the local context are critical to success [20]. Our results follow from the literature mentioned in the Background section and show that there are large differences in HTA institutionalization and resources across countries. The results show that HICs report much higher rates of having legislative requirements, having allocated public sector budgets, and having guidelines available for assessment.

The survey can be used to identify areas for improvement across all country income groups – for example our results show that broad stakeholder participation is not common and those wishing to expand participation can use these results to identify where other countries are doing this. In addition, while there are many countries that have processes in place for managing conflict of interest, appeals processes are much less common. While there has been work to understand good practice guidelines for HTA over the years, more is needed on links with actual decision-making and where HTA processes can be the most helpful [21], [22]. Finally, developing awareness of the importance of HTA requires that the value in terms of the health and economic benefits given the investments needed is recognized [23].

This survey is the largest of its kind in terms of the number of countries included. The survey covered a broad range of countries from multiple regions. The survey also allowed for a broad definition of decision-making processes beyond just HTA, and one of its strengths is capturing the characteristics of decision-making processes across many countries that do not call the process “HTA”, however, we do note that this runs the risk of oversimplifying a wide range of complex processes. We found that “HTA” was the only adequate term for encompassing these processes, though it was used as a summary term only after the data was collected. In the future, a better system of terminology for describing these processes may need to be developed. A limitation of the survey is that country HTA and decision-making processes are highly contextualized and providing a full picture of their nuances requires further investigation. Many questions that have a “yes/no” or multiple-choice formats contain a lot of details that underpin the responses, and it will be helpful in the future to unpack the rationale for responses further. There were also many questions with high non-response rates, which are shown in the results. Another limitation of this work is the lack of external validation. Given that the responses were provided in large part by government officials, the responses were accepted as provided by the respondent. Despite this, the responses were checked for outliers or non-sensical information and follow-up with respondents was undertaken when necessary.

In future survey rounds, questions can be asked to show the importance of regional networks, which can be seen by the important role that REDETSA (Red de Evaluación de Tecnologías en Salud de los Américas) has played in the PAHO region – fostering institutionalization by bringing together stakeholders and providing a platform for sharing evidence and knowledge [10]. Regional networks have also been developed in Europe and Asia with EuNetHTA (European Network for Health Technology Assessment) and HTAsiaLink and international networks have been created with INAHTA (International Network for Agencies of Health Technology Assessment) and HTAi (Health Technology Assessment International). Another future area of research is to identify how HTA, and other decision-making processes fit into different health financing models. HTA processes are typically associated with single-payer public health systems although there are plenty of examples where they exist in pluralistic health systems [24]. Finally, future survey rounds can show changes over time. For example, despite the fact that a previous survey was conducted in 2015, that fact that there were different respondents and different questionnaires meant that there was limited scope for comparison across the two datasets [14].

## 5 Conclusion

Using a five-part framework of HTA institutionalization, the results show that while many countries have HTA bodies in place, these serve different functions. The findings also show differences in the development of HTA processes by country income levels according to elements such as whether legislative requirements are in place whether there is an allocated public sector budgets and whether guidelines exist. This highlights that there are likely more established and sustainable processes in higher income country groups. Developed processes in HICs also show room for improvement in areas such as social participation and appeal. These results give a picture that there is much further progress to be made in HTA globally, especially in low- and middle-income countries. Lack of awareness of the importance of HTA and institutionalization were the top two barriers to HTA utilization and lack of budgets, data and human resources featured as the top barriers to HTA production. Mitigating these barriers is not easy and requires concerted efforts and adequate resources.

## CRediT authorship contribution statement

**Andrew J. Mirelman:** Writing – review & editing, Writing – original draft, Visualization, Validation, Supervision, Software, Resources, Project administration, Methodology, Investigation, Formal analysis, Data curation, Conceptualization. **Kratu Goel:** Writing – review & editing, Writing – original draft, Validation, Software, Project administration, Formal analysis, Data curation, Conceptualization. **Tessa Tan-Torres Edejer:** Writing – review & editing, Writing – original draft, Supervision, Methodology, Conceptualization.

## Funding

This research did not receive any specific grant from funding agencies in the public, commercial, or not-for-profit sectors.

## Declaration of competing interest

The authors declare that they have no known competing financial interests or personal relationships that could have appeared to influence the work reported in this paper.
