# Supplementary material for: The global landscape of country-level health technology assessment processes: A survey among 104 countries
Source: Health Policy Open. 2025 Mar 27;8:100138. doi: 10.1016/j.hpopen.2025.100138 (PMC11999493; doi:10.1016/j.hpopen.2025.100138)
Supplement: Supplementary Data 1 [file mmc1.docx]

Annex

List of 10 countries where survey piloting was conducted: Argentina, China, Ethiopia, Malawi, Norway, Philippines, Spain, Sweden, Thailand, and Tunisia.
