# Supplementary material for: The global landscape of country-level health technology assessment processes: A survey among 104 countries
Source: Health Policy Open. 2025 Mar 27;8:100138. doi: 10.1016/j.hpopen.2025.100138 (PMC11999493; doi:10.1016/j.hpopen.2025.100138)
Supplement: Supplementary Data 2 [file mmc2.docx]

# Annex

## Barriers to HTA Production

For the barriers to HTA production, ‘budget availability’ and ‘dedicated human resources’ were almost equally likely to be the top ranked option. ‘Data availability’ was also sometimes listed as a top-ranked barrier here. By income group, ‘budget availability’ was seen to feature as a prominent barrier in LMICs and UMICs; while in HICs, ‘data availability’ and ‘dedicated human resources’ were the top ranked barriers. ‘Knowledge of methods’ was also a common top-ranked barrier in LMICs’ and in all groups, sometimes in the top three as well.

## Training Needs

In terms of training needs, countries were asked to rank three needs for educational development. 41 (39%) of countries ranked ‘higher education’ as the top need for further development. ‘Internal staff training’ and ‘courses/seminars’ were the top-ranked training need for 25 (25%) and 18 (18%) of countries, respectively. By income group, higher education was the most commonly top ranked barrier for HICs and UMICs while internal staff training was the most commonly top-ranked barrier in LMICs and LICs.
