# Supplementary material for: The global landscape of country-level health technology assessment processes: A survey among 104 countries
Source: Health Policy Open. 2025 Mar 27;8:100138. doi: 10.1016/j.hpopen.2025.100138 (PMC11999493; doi:10.1016/j.hpopen.2025.100138)
Supplement: Supplementary Data 3 [file mmc3.docx]

**Table 2A. Overall Results to Selected Survey Questions by World Bank Income Grouping**

|  |  | **Total** | | | | | **HIC** | | | | **UMIC** | | | | **LMIC** | | | | **LIC** | | | |
| --- | --- | --- | --- | --- | --- | --- | --- | --- | --- | --- | --- | --- | --- | --- | --- | --- | --- | --- | --- | --- | --- | --- |
| **Variable** | **Response option (when applicable)** | **Yes** | | **No** | **I don’t know** | **N/R** | **Yes** | **No** | **I don’t know** | **N/R** | **Yes** | **No** | **I don’t know** | **N/R** | **Yes** | **No** | **I don’t know** | **N/R** | **Yes** | **No** | **I don’t know** | **N/R** |
| **Mandate and Legal Framework** | | | | | | | | | | | | | | | | | | | | | | |
| **Functions** | **Planning and budgeting** | 81 | 19 | | N/A | 4 | 24 | 9 | N/A | 3 | 21 | 4 | N/A | 0 | 25 | 4 | N/A | 1 | 11 | 2 | N/A | 0 |
|  | **Clinical practice guidelines** | 78 | 21 | | N/A | 5 | 24 | 9 | N/A | 3 | 20 | 5 | N/A | 0 | 23 | 5 | N/A | 2 | 11 | 2 | N/A | 0 |
|  | **Design of Health Benefit Packages** | 66 | 34 | | N/A | 4 | 22 | 12 | N/A | 2 | 16 | 9 | N/A | 0 | 20 | 8 | N/A | 2 | 8 | 5 | N/A | 0 |
|  | **Protocols for public health programmes** | 59 | 38 | | N/A | 7 | 18 | 13 | N/A | 5 | 14 | 11 | N/A | 0 | 17 | 11 | N/A | 2 | 10 | 3 | N/A | 0 |
|  | **Public Procurement of Medicines** | 56 | 45 | | N/A | 3 | 18 | 15 | N/A | 3 | 9 | 16 | N/A | 0 | 20 | 10 | N/A | 0 | 9 | 4 | N/A | 0 |
|  | **Indicators of quality of care** | 56 | 43 | | N/A | 5 | 19 | 14 | N/A | 3 | 11 | 14 | N/A | 0 | 15 | 13 | N/A | 2 | 11 | 2 | N/A | 0 |
|  | **Pricing/pricing negotiations** | 54 | 48 | | N/A | 2 | 26 | 10 | N/A | 0 | 11 | 14 | N/A | 0 | 12 | 16 | N/A | 2 | 5 | 8 | N/A | 0 |
|  | **Objectives for P4P schemes** | 31 | 66 | | N/A | 7 | 11 | 20 | N/A | 5 | 6 | 19 | N/A | 0 | 10 | 18 | N/A | 2 | 4 | 9 | N/A | 0 |
|  | **Other** | 19 | 0 | | N/A | 85 | 7 | 0 | N/A | 29 | 5 | 0 | N/A | 20 | 4 | 0 | N/A | 26 | 3 | 0 | N/A | 10 |
| **Legislative requirement** |  | 54 | 34 | | 5 | 11 | 24 | 6 | 1 | 5 | 15 | 6 | 2 | 2 | 9 | 17 | 2 | 2 | 6 | 5 | 0 | 2 |
| **Binding results** |  | 32 | 17 | | 2 | 53 | 15 | 7 | 0 | 14 | 6 | 8 | 0 | 11 | 7 | 2 | 0 | 21 | 4 | 0 | 2 | 7 |
| **Institutional Arrangements** | | | | | | | | | | | | | | | | | | | | | | |
| **Collaboration within the country (*n=67)** | **Ministries or other Government Institutions** | 54 | 11 | | N/A | 2 | 19 | 4 | N/A | 0 | 10 | 4 | N/A | 0 | 16 | 3 | N/A | 1 | 9 | 1 | N/A | 0 |
|  | **Academia/University** | 46 | 19 | | N/A | 2 | 11 | 11 | N/A | 1 | 11 | 3 | N/A | 0 | 17 | 2 | N/A | 1 | 7 | 3 | N/A | 0 |
|  | **Professional associations** | 37 | 29 | | N/A | 1 | 9 | 13 | N/A | 1 | 7 | 7 | N/A | 0 | 14 | 6 | N/A | 0 | 7 | 3 | N/A | 0 |
|  | **Hospital** | 31 | 32 | | N/A | 4 | 6 | 14 | N/A | 3 | 9 | 4 | N/A | 1 | 10 | 10 | N/A | 0 | 6 | 4 | N/A | 0 |
|  | **Patient Associations** | 21 | 42 | | N/A | 4 | 9 | 13 | N/A | 1 | 3 | 10 | N/A | 1 | 8 | 11 | N/A | 1 | 1 | 8 | N/A | 1 |
|  | **Industry** | 17 | 44 | | N/A | 6 | 7 | 13 | N/A | 3 | 4 | 9 | N/A | 1 | 5 | 14 | N/A | 1 | 1 | 8 | N/A | 1 |
|  | **Other** | 19 | 42 | | N/A | 6 | 9 | 12 | N/A | 2 | 5 | 8 | N/A | 1 | 1 | 17 | N/A | 2 | 4 | 5 | N/A | 1 |
| **Collaboration outside the country (*n=53)** | **Ministries or other Government Institutions** | 28 | 22 | | N/A | 3 | 10 | 8 | N/A | 1 | 6 | 7 | N/A | 0 | 9 | 5 | N/A | 1 | 3 | 2 | N/A | 1 |
|  | **Academia/University** | 18 | 30 | | N/A | 5 | 4 | 13 | N/A | 2 | 6 | 6 | N/A | 1 | 7 | 7 | N/A | 1 | 1 | 4 | N/A | 1 |
|  | **Professional associations** | 8 | 39 | | N/A | 6 | 2 | 15 | N/A | 2 | 2 | 10 | N/A | 1 | 3 | 10 | N/A | 2 | 1 | 4 | N/A | 1 |
|  | **Hospital** | 3 | 44 | | N/A | 6 | 1 | 16 | N/A | 2 | 0 | 12 | N/A | 1 | 0 | 13 | N/A | 2 | 2 | 3 | N/A | 1 |
|  | **Industry** | 2 | 45 | | N/A | 6 | 1 | 16 | N/A | 2 | 0 | 12 | N/A | 1 | 0 | 13 | N/A | 2 | 1 | 4 | N/A | 1 |
|  | **Patient Associations** | 1 | 46 | | N/A | 6 | 1 | 16 | N/A | 2 | 0 | 12 | N/A | 1 | 0 | 13 | N/A | 2 | 0 | 5 | N/A | 1 |
|  | **Other** | 27 | 24 | | N/A | 2 | 11 | 8 | N/A | 0 | 8 | 4 | N/A | 1 | 4 | 10 | N/A | 1 | 4 | 2 | N/A | 0 |
| **Standard Methodology/Process Guideline** |  | 52 | 39 | | N/A | 13 | 26 | 4 | N/A | 6 | 15 | 7 | N/A | 3 | 7 | 20 | N/A | 3 | 4 | 8 | N/A | 1 |
| **Mechanism for translation** |  | 41 | 37 | | N/A | 26 | 13 | 12 | N/A | 11 | 9 | 11 | N/A | 5 | 13 | 11 | N/A | 6 | 6 | 3 | N/A | 4 |
| **Provision for rapid assessment - Non-Emergency** |  | 50 | 32 | | N/A | 22 | 17 | 12 | N/A | 7 | 13 | 5 | N/A | 7 | 14 | 11 | N/A | 5 | 6 | 4 | N/A | 3 |
| **Provision for rapid assessment - Emergency** |  | 52 | 28 | | N/A | 24 | 15 | 12 | N/A | 9 | 12 | 2 | N/A | 11 | 17 | 10 | N/A | 3 | 8 | 4 | N/A | 1 |
| **Allocated public-sector budget** |  | 59 | 33 | | 5 | 7 | 27 | 6 | 0 | 3 | 12 | 10 | 1 | 2 | 16 | 13 | 1 | 0 | 4 | 4 | 3 | 2 |
| **Private Funding** |  | 15 | 62 | | N/A | 27 | 6 | 24 | N/A | 6 | 2 | 11 | N/A | 12 | 6 | 20 | N/A | 4 | 1 | 7 | N/A | 5 |
| **Procedural aspects of Assessment, Appraisal and Recommendation** | | | | | | | | | | | | | | | | | | | | | | |
| **Economic evaluation guidelines** |  | 41 | 43 | | N/A | 20 | 22 | 9 | N/A | 5 | 12 | 6 | N/A | 7 | 5 | 21 | N/A | 4 | 2 | 7 | N/A | 4 |
| **Officially endorsed Cost-Effectiveness Threshold** |  | 19 | 63 | | N/A | 22 | 11 | 20 | N/A | 5 | 5 | 10 | N/A | 10 | 2 | 23 | N/A | 5 | 1 | 10 | N/A | 2 |
| **Variation in threshold (*n=19)** |  | 8 | 10 | | N/A | 1 | 6 | 4 | N/A | 1 | 1 | 4 | N/A | 0 | 0 | 2 | N/A | 0 | 1 | 0 | N/A | 0 |
| **Stakeholders have equal voice in Appraisal** |  | 55 | 14 | | N/A | 35 | 19 | 6 | N/A | 11 | 11 | 2 | N/A | 12 | 17 | 5 | N/A | 8 | 8 | 1 | N/A | 4 |
| **Members of Appraisal body provide a conflict of interest** |  | 60 | 16 | | N/A | 28 | 23 | 3 | N/A | 10 | 19 | 1 | N/A | 5 | 13 | 8 | N/A | 9 | 5 | 4 | N/A | 4 |
| **Non-represented stakeholders allowed to react** |  | 42 | 30 | | N/A | 32 | 14 | 11 | N/A | 11 | 13 | 6 | N/A | 6 | 12 | 6 | N/A | 12 | 3 | 7 | N/A | 3 |
| **Separate entity for Recommendation** |  | 48 | 56 | | N/A | 0 | 20 | 16 | N/A | 0 | 10 | 15 | N/A | 0 | 13 | 17 | N/A | 0 | 5 | 8 | N/A | 0 |
| **Possibility of Appeal** |  | 22 | 11 | | N/A | 71 | 9 | 4 | N/A | 23 | 4 | 2 | N/A | 19 | 6 | 4 | N/A | 20 | 3 | 1 | N/A | 9 |
| **Publication of Recommendations** | **Minutes of the meetings** | 29 | 68 | | N/A | 7 | 14 | 18 | N/A | 4 | 1 | 23 | N/A | 1 | 11 | 18 | N/A | 1 | 3 | 9 | N/A | 1 |
|  | **Assessment reports** | 48 | 53 | | N/A | 3 | 22 | 14 | N/A | 0 | 8 | 17 | N/A | 0 | 16 | 12 | N/A | 2 | 2 | 10 | N/A | 1 |
|  | **Recommendations (or decisions where relevant)** | 48 | 54 | | N/A | 2 | 21 | 14 | N/A | 1 | 9 | 16 | N/A | 0 | 15 | 15 | N/A | 0 | 3 | 9 | N/A | 1 |
|  | **Rationale for the decision** | 27 | 72 | | N/A | 5 | 12 | 21 | N/A | 3 | 4 | 20 | N/A | 1 | 9 | 21 | N/A | 0 | 2 | 10 | N/A | 1 |
|  | **Others** | 12 | 82 | | N/A | 10 | 5 | 25 | N/A | 6 | 3 | 21 | N/A | 1 | 3 | 25 | N/A | 2 | 1 | 11 | N/A | 1 |
|  | **No outputs from the recommendation process are published** | 16 | 77 | | N/A | 11 | 4 | 25 | N/A | 7 | 4 | 20 | N/A | 1 | 4 | 24 | N/A | 2 | 4 | 8 | N/A | 1 |
| **Monitoring and Evaluation** | | | | | | | | | | | | | | | | | | | | | | |
| **Indicators to assess impact** |  | 32 | 45 | | N/A | 27 | 13 | 17 | N/A | 6 | 6 | 7 | N/A | 12 | 7 | 16 | N/A | 7 | 6 | 5 | N/A | 2 |
| **Criteria to assess impact (*n=32)** | **Health outcomes** | 24 | 6 | | N/A | 2 | 8 | 4 | N/A | 1 | 3 | 2 | N/A | 1 | 7 | 0 | N/A | 0 | 6 | 0 | N/A | 0 |
|  | **Variation in practice (before/after)** | 22 | 8 | | N/A | 2 | 8 | 4 | N/A | 1 | 1 | 4 | N/A | 1 | 5 | 2 | N/A | 0 | 4 | 1 | N/A | 1 |
|  | **Cost of medical practice** | 19 | 12 | | N/A | 1 | 9 | 4 | N/A | 0 | 3 | 3 | N/A | 0 | 3 | 4 | N/A | 0 | 4 | 1 | N/A | 1 |
|  | **Changes in health from patient view** | 19 | 10 | | N/A | 3 | 7 | 4 | N/A | 2 | 3 | 2 | N/A | 1 | 5 | 2 | N/A | 0 | 4 | 2 | N/A | 0 |
|  | **Variation in practice (current/recommended)** | 18 | 11 | | N/A | 3 | 9 | 3 | N/A | 1 | 2 | 3 | N/A | 1 | 5 | 2 | N/A | 0 | 6 | 0 | N/A | 0 |
|  | **Level of technology diffusion** | 16 | 14 | | N/A | 2 | 8 | 4 | N/A | 1 | 1 | 5 | N/A | 0 | 2 | 5 | N/A | 0 | 5 | 0 | N/A | 1 |
|  | **Changes within organizations or facilities** | 14 | 15 | | N/A | 3 | 4 | 7 | N/A | 2 | 1 | 4 | N/A | 1 | 5 | 2 | N/A | 0 | 4 | 2 | N/A | 0 |
|  | **Changes in the law** | 10 | 19 | | N/A | 3 | 4 | 8 | N/A | 1 | 1 | 4 | N/A | 1 | 2 | 5 | N/A | 0 | 3 | 2 | N/A | 1 |
|  | **Other** | 3 | 0 | | N/A | 29 | 3 | 0 | N/A | 10 | 0 | 0 | N/A | 6 | 0 | 0 | N/A | 7 | 0 | 0 | N/A | 6 |
