# Supplementary material for: The global landscape of country-level health technology assessment processes: A survey among 104 countries
Source: Health Policy Open. 2025 Mar 27;8:100138. doi: 10.1016/j.hpopen.2025.100138 (PMC11999493; doi:10.1016/j.hpopen.2025.100138)
Supplement: Supplementary Data 4 [file mmc4.docx]

WHO Disclaimer on terminology: The term “countries” or “national” should be understood to refer to countries and areas. The designations employed and the presentation of the material in this platform do not imply the expression of any opinion whatsoever on the part of WHO concerning the legal status of any country, territory, city, or area or of its authorities, or concerning the delimitation of its frontiers or boundaries.
