# Supplementary material for: The global landscape of country-level health technology assessment processes: A survey among 104 countries
Source: Health Policy Open. 2025 Mar 27;8:100138. doi: 10.1016/j.hpopen.2025.100138 (PMC11999493; doi:10.1016/j.hpopen.2025.100138)
Supplement: Supplementary Data 5 [file mmc5.docx]

# Annexes

## Sources from which survey respondents were identified

Figure A1: Sources of Survey Respondents

| **Nomination Category** | ***Definition*** |
| --- | --- |
| Officially nominated | Have receipt of letter from MOH (e.g., Burkina Faso), OR nomination was received through process of sending letters to WRs in 2020/2021 OR respondent has nomination letter confirmed from 2015 survey round OR name provided by email from MOH representative (in 2015 or 2021 round) |
| Officially acknowledged | MOH or representative body acknowledges that agency or entity will fill out the survey |
| HQ identified | Name identified by HQ team |
| RO identified | Name identified by RO team |
| WCO identified | Name provided by WCO team |
| 2015 respondent - unofficial | 2015 respondent (unable to find official nomination) |
| Consultant | Consultant contracted to respond to survey |
| WCO respondent | WCO team member responds to survey |

Table A1: Nomination categories of survey respondents

## Survey Translation and Data Cleaning

A PDF copy of each final response was saved in the WHO SharePoint folder for future referencing and validation with respondents.

Translation of responses was done using Google translate and French and Spanish responses were reviewed by people proficient in those languages. Arabic and Russian responses were reviewed by native and proficient speakers in the EMRO and EURO regional office of WHO. Translated results were transferred back to the clean data sheet.

The process of data cleaning involved downloading the data in excel from the Dataform software and linking each submitted response with their unique response ID. All columns and variables were standardized to reflect the questions from the survey. Variables were then standardized as “Yes”, “No”, “N/A” or “N/R” as applicable, and external information was appended to the dataset for all WHO member states for items such as income-group (World Bank), region, Population (in thousands) 2018, Current Health Expenditure (CHE) per capita in US$ 2018, and ISO3 code^[[1]](#footnote-1)^. A new variable for “Pharmacist” was added to the list of occupations for respondents. In multiple choice questions, the data was output as 1’s and 0’s, therefore, all 1’s were converted to “Yes” and all 0’s were converted to “No”.

## Functions of decision-making process

| Functions | Yes | Process is called HTA | Process is not called HTA |
| --- | --- | --- | --- |
| Planning and budgeting | 81 | 47 | 34 |
| Clinical practice guidelines | 78 | 49 | 29 |
| Design of Health Benefit Packages | 66 | 43 | 23 |
| Protocols for public health programmes | 59 | 34 | 25 |
| Public Procurement of Medicines | 56 | 33 | 23 |
| Indicators of quality of care | 56 | 30 | 26 |
| Pricing/pricing negotiations of medical technologies | 54 | 40 | 14 |
| Objectives for pay-for-performance schemes | 31 | 19 | 12 |
| Other | 18 | 13 | 5 |

Table A2: Breakdown of functions of decision-making process on the basis of whether or not the process is called HTA

1. Source for Current Health Expenditure (CHE) per capita in US$ 2018 and Population (in thousands) 2018 is <https://apps.who.int/nha/database/ViewData/Indicators/en> [↑](#footnote-ref-1)
