# Supplementary material for: The global landscape of country-level health technology assessment processes: A survey among 104 countries
Source: Health Policy Open. 2025 Mar 27;8:100138. doi: 10.1016/j.hpopen.2025.100138 (PMC11999493; doi:10.1016/j.hpopen.2025.100138)
Supplement: Supplementary Data 6 [file mmc6.docx]

Health Technology Assessment Survey 2020

Dear Respondent,

In response to the World Health Assembly Resolution 67.23, a baseline Global Survey was completed in 2015 which enabled the WHO Secretariat to assess the status of Health Technology Assessment (HTA) in Member

[States. The results of the 2015 Global Survey can be accessed in the Global Report](https://www.who.int/health-technology-assessment/MD_HTA_oct2015_final_web2.pdf?ua=1)

[(https://www.who.int/health-technology-assessment/MD_HTA_oct2015_final_web2.pdf?ua=1). WHO is now](https://www.who.int/health-technology-assessment/MD_HTA_oct2015_final_web2.pdf?ua=1) seeking to update the results of this survey, by collecting current information about the status of HTA processes in Member States. **For background information about the WHA** [**resolution and the framework underlying this survey, please review this presentation** - [Introduction - CLICK HERE (https://www.slideshare.net/secret/L6IUSguXHrpY9T)].](https://www.slideshare.net/secret/L6IUSguXHrpY9T)

Filling out this survey is estimated to take approximately 60 minutes of your valuable time. This survey has a content sections asking about HTA processes and barriers, and at the very end we also ask for your authorization to use the information you have provided.

**The questions can all be answered by the same person or answered in a group. We recommend that you utilize multiple people for these responses if you don't feel like you can address a section accurately.** You can stop the survey at any time and save your answers at which time you’ll be asked to create a log-in. If needed, you may forward the link and log-in information to other respondents who might be better suited to respond to a section. Please note that once the survey has been submitted it is no longer possible to modify the answers unless you contact us directly. **A more detailed** [**presentation on completing this survey is available here [How to Answer - CLICK HERE (https://www.slideshare.net/secret/HOkV42lG9W0osj)].**](https://www.slideshare.net/secret/HOkV42lG9W0osj)

Your effort is very important to guide the WHO Secretariat and other partners regarding future planning and development of HTA guidelines and benchmarks, as well as to ensure that current information is

available in your [country’s profile (https://www.who.int/health-technology-assessment/country-profile/en/)](https://www.who.int/health-technology-assessment/country-profile/en/). A

synthesis of the responses to this survey will also be made available in the survey report.

[If you need assistance or have any concerns, you can contact **htechassessment@who.int**](mailto:htechassessment@who.int)

[**(mailto:htechassessment@who.int)**. The deadline to submit the survey is 15^th^ November 2020.](mailto:htechassessment@who.int)

Thank you very much!

1. NOTE: For the purpose of this survey medical devices include in-vitro diagnostics and laboratory

equipment, imaging equipment, single use devices, and assistive devices.

Respondent Information

1 Please provide the following information - *

2 **What is your primary professional background?**

Please choose **all** that apply:

Economist Statistician

Public Health Specialist Medical Degree

Other clinical qualification Epidemiologist Administration/Planning

Other:

**You may select a maximum of 2 options.**

3

**What is your primary organization or affiliation?**

Please choose all that apply and provide a comment: Government - Ministry of Health

Other Government Ministry/Agency

International not-for-profit organization (iNGO)

National or Sub-national not-for-profit organization (NGO)

International Organization (incl. United Nations)

Consultancy firm/consultant

Academic Institution

Other

**You MUST specify/give full name of your organisation.**

**ation**

4

**If we should want to contact you after the survey has been completed, what is your preferred method of contact?**

Please choose all that apply and provide a comment: E-mail

Phone

Skype

***Please provide a response in the box accompanying the option selected***

|  | **First Name** | **Last Name** | **Organization** | **Position** | **Email** | **Survey Sections Filled** |
| --- | --- | --- | --- | --- | --- | --- |
| **Contributor 1** |  |  |  |  |  |  |
| **Contributor 2** |  |  |  |  |  |  |
| **Contributor 3** |  |  |  |  |  |  |
| **Contributor 4** |  |  |  |  |  |  |
| **Contributor 5** |  |  |  |  |  |  |
| **Contributor 6** |  |  |  |  |  |  |

HTA Institutions and Governance

5

**If you have collaborated with other people to answer this survey, please list below the contact information and affiliation of all individuals who contributed.**

6 **Does your country have a systematic formal process by which information is gathered to support healthcare decision-making?** *

Please choose **all** that apply:

Yes, at the national level Yes, at the subnational level None

**NOTE: The process can be within a committee, unit, department or an established HTA organization at a national or subnational level.**

**Decision-making here refers to a process for inclusion in Health benefit packages for provision or for reimbursement of health services**

**Please answer the remainder of the survey for the national level process. If you do not have a national level**

**process, only then answer for the subnational level process. If you selected None, please proceed to the next question.**

**Subnational Scheme refers to a regional or state level scheme.**

7 **Since you answered ‘No’ to having a systematic formal process for information gathering to support healthcare decision-making, we will now ask a few questions about any processes that you do have in place (please note, that you only need to answer to the best degree possible. If there are no processes in place at all, please say so).**

Only answer this question if the following conditions are met:

Answer was 'None' at question '6 [HTAIG1]' (Does your country have a systematic formal process by which information is gathered to support healthcare decision-making?)

8 **Please describe your health-care decision making process in the box below.**

Only answer this question if the following conditions are met:

Answer was 'None' at question '6 [HTAIG1]' (Does your country have a systematic formal process by which information is gathered to support healthcare decision-making?)

Please write your answer here:

10

**What entities(e.g. organizations/individuals/officials) are involved in any decisions regarding inclusion or exclusion of health technologies and interventions? For each, please state what functions they perform? Functions could include: identifying interventions, assessing evidence, appraising evidence, and making recommendations.**

Only answer this question if the following conditions are met:

Answer was 'None' at question '6 [HTAIG1]' (Does your country have a systematic formal process by which information is gathered to support healthcare decision-making?)


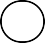


9

**Can you indicate whether there are plans for implementing a systematic formal process and if so, what that time-frame is?**

Only answer this question if the following conditions are met:

Answer was 'None' at question '6 [HTAIG1]' (Does your country have a systematic formal process by which information is gathered to support healthcare decision-making?)

Please choose **only one** of the following:

There is no plan to implement such a process


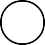
 There will be a process in place in less than 1 year
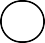
 There will be a process in place in 1-2 years


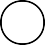
 There will be a process in place in 3-5 years


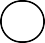
 There will be a process in place in more than 5 years.

|  | **Title of Entity** | **Functions Performed** |
| --- | --- | --- |
| **Entity1** |  |  |
| **Entity 2** |  |  |
| **Entity 3** |  |  |
| **Entity 4** |  |  |
| **Entity 5** |  |  |


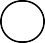


11

**Are any prioritization criteria used to determine which health technologies and interventions are considered in government health schemes in the country? If yes, please describe them in the box.**

Only answer this question if the following conditions are met:

Answer was 'None' at question '6 [HTAIG1]' (Does your country have a systematic formal process by which information is gathered to support healthcare decision-making?)

Please choose **only one** of the following:

Yes
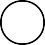
 No

Make a comment on your choice here:


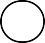


12

**Is evidence collected for making decisions about the inclusion or exclusion of health technologies and interventions? Please note, this could include translating evidence from other settings. If yes, please provide details in the comment box.**

Only answer this question if the following conditions are met:

Answer was at question '6 [HTAIG1]' (Does your country have a systematic formal process by which information is gathered to support healthcare decision-making?)

Please choose **only one** of the following:

Yes
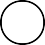
 No

Make a comment on your choice here:


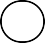


13

**Are there any formal or informal procedures, rules, thresholds, or laws that factor in to the decision-making related to health technologies and interventions? If yes, please provide details in the comment box.**

Only answer this question if the following conditions are met:

Answer was 'None' at question '6 [HTAIG1]' (Does your country have a systematic formal process by which information is gathered to support healthcare decision-making?)

Please choose **only one** of the following:

Yes
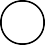
 No

Make a comment on your choice here:


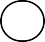


14

**Are there any provisions for revising decisions about health technologies and interventions once they are made? If yes, please provide details in the comment box.**

Only answer this question if the following conditions are met:

Answer was 'None' at question '6 [HTAIG1]' (Does your country have a systematic formal process by which information is gathered to support healthcare decision-making?)

Please choose **only one** of the following:

Yes
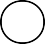
 No

Make a comment on your choice here:

15 **For which of the following functions is information gathered in your setting?**

Please choose **all** that apply:

Clinical practice guidelines Planning and budgeting

Pricing/pricing negotiations of medical technologies Indicators of quality of care

To determine objectives for pay-for-performance schemes Design of Health Benefit Packages

Public Procurement of Medicines Protocols for public health programmes

Other:


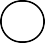


16 **Do you refer to this process as Health Technology Assessment (HTA)?**

Only answer this question if the following conditions are met:

Answer was 'Yes, at the subnational level' *or* 'Yes, at the national level' at question '6 [HTAIG1]' (Does your country have a systematic formal process by which information is gathered to support healthcare decision-making?)

Please choose **only one** of the following:

Yes
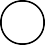
 No


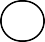


17 **Do you have a standard methodology or process guideline for conducting HTA or decision-making process? If yes, please provide a link to the document in the comment box or upload the document in the following question.**

Only answer this question if the following conditions are met:

Answer was 'Yes, at the subnational level' *or* 'Yes, at the national level' at question '6 [HTAIG1]' (Does your country have a systematic formal process by which information is gathered to support healthcare decision-making?)

Please choose **only one** of the following:

Yes
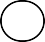
 No

Make a comment on your choice here:

**We refer to the broader HTA process and specific questions about methods for economic evaluation (e.g. cost-effectiveness) will be asked later.**

**eutical/**

**es**

**c**

19

**For each of the following areas of the HTA or decision-making process, please list the organisation(s) or entity(ies) responsible for the components of assessment, appraisal and recommendation for each category of interventions?**

Only answer this question if the following conditions are met:

Answer was 'Yes, at the national level' *or* 'Yes, at the subnational level' at question '6 [HTAIG1]' (Does your country have a systematic formal process by which information is gathered to support healthcare decision-making?)

**ns**

*Please add multiple entities to each box as needed.*

*Population Level Health Interventions - (i.e. large scale screening/vaccination programs)*

18 If possible, please upload the standard HTA methodology/guidance document here.

Only answer this question if the following conditions are met:

Answer was 'Yes' at question '17 [HTAIG4a]' (Do you have a standard methodology or process guideline for conducting HTA or decision-making process? If yes, please provide a link to the document in the comment box or upload the document in the following question.)

Kindly attach the aforementioned documents along with the survey

|  | **Pharmac Medicines** | **Medical Procedur** | **Medical Devices** | **Diagnosti Tests** | **Population Level**  **Health Interventio** |
| --- | --- | --- | --- | --- | --- |
| **Assessment (Data)** |  |  |  |  |  |
| **Appraisal (Dialogue)** |  |  |  |  |  |
| **Recommendation (Decision)** |  |  |  |  |  |


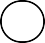


20 **Is there a legislative and/or regulatory requirement to consider the results of HTA or decision-making process while making coverage decisions or inclusion of an intervention in the health benefits package?**

Only answer this question if the following conditions are met:

Answer was 'Yes, at the subnational level' *or* 'Yes, at the national level' at question '6 [HTAIG1]' (Does your country have a systematic formal process by which information is gathered to support healthcare decision-making?)

Please choose **only one** of the following:

Yes
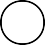
 No


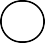
 I don't know

21

**Please specify the institutional mechanisms (e.g. law, or regulation) and/or provide a link to the appropriate document.**

**You may also upload any relevant documents in the following question.**

Only answer this question if the following conditions are met:

Answer was 'Yes' at question '20 [HTAIG5]' (Is there a legislative and/or regulatory requirement to consider the results of HTA or decision-making process while making coverage decisions or inclusion of an intervention in the health benefits package? )

Please write your answer here:

22 Please upload any relevant documents if you wish to do so

Only answer this question if the following conditions are met:

Answer was 'Yes' at question '20 [HTAIG5]' (Is there a legislative and/or regulatory requirement to consider the results of HTA or decision-making process while making coverage decisions or inclusion of an intervention in the health benefits package? )

Kindly attach the aforementioned documents along with the survey


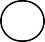


23

**Are the results of the HTA or decision-making process considered binding by law?**

Only answer this question if the following conditions are met:

Answer was 'Yes' at question '20 [HTAIG5]' (Is there a legislative and/or regulatory requirement to consider the results of HTA or decision-making process while making coverage decisions or inclusion of an intervention in the health benefits package? )

Please choose **only one** of the following:

Yes
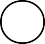
 No


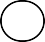
 I don't know


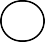


24 **Has your organization formally collaborated with or is it currently collaborating with other organizations on the HTA or decision-making process?**

Please choose **only one** of the following:

Yes, with organizations within our country
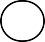
 Yes, with organization from other countries
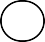
 Both


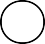
 No

***Formal Collaboration**

- 1. **Which kind of organizations from YOUR COUNTRY have you been collaborating with?**

##### Only answer this question if the following conditions are met:

Answer was 'Yes, with organizations within our country' *or* 'Both' at question '24 [HTAIG10]' (Has your organization formally collaborated with or is it currently collaborating with other organizations on the HTA or decision-making process?)

Please choose all that apply and provide a comment: Ministries or other Government Institutions

Academia/University

Professional associations

Hospital

Industry

Patient Associations

Other, please specify

**Please refer to only formal collaborations**

**Please give name/details of the Organisation(s) that you select**

**If you have a website with a list of institutions, please provide the link in the Other box**

**You can select multiple options**

- 1. **Which kind of organizations from OTHER COUNTRIES have you been collaborating with?**

##### Only answer this question if the following conditions are met:

Answer was 'Both' *or* 'Yes, with organization from other countries' at question '24 [HTAIG10]' (Has your organization formally collaborated with or is it currently collaborating with other organizations on the HTA or decision-making process?)

Please choose all that apply and provide a comment: Ministries or other Government Institutions

Academia/University

Professional associations

Hospital

Industry

Patient Associations

Other, please specify

Please refer to only formal collaborations

Please give name/details of the Organisation(s) that you select

If you have a website with a list of institutions, please provide the link in the Other box You can select multiple options

27 **Who is responsible for nominating interventions to be reviewed and selecting the scope of priorities for the assessment and appraisal stages?**

Only answer this question if the following conditions are met:

Answer was 'Yes, at the national level' *or* 'Yes, at the subnational level' at question '6 [HTAIG1]' (Does your country have a systematic formal process by which information is gathered to support healthcare decision-making?)

Please choose **all** that apply:

Scientific Committee of the HTA or decision-making body Executive Board of the HTA or decision-making body Director of the HTA or decision-making body Department/Ministry of Health

National Health Service Patient Organizations Civil Society

Other:

**Please specify the entity in the box if selecting Other You can select multiple options**


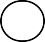


28 **Does your HTA or decision-making process include mechanisms for translation and/or contextualization of evidence from other settings? If Yes, can you please describe this process further in the comment box?**

Please choose **only one** of the following:

Yes
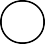
 No

Make a comment on your choice here:


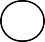


29 **Does your HTA or health decision-making process include any provisions for rapidly assessing evidence, appraising evidence, and making decisions in a non-emergency context?**

Please choose **only one** of the following:

Yes
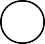
 No

30 **Can you please describe what criteria are used to qualify something for this “rapid” process?**

Only answer this question if the following conditions are met:

Answer was 'Yes' at question '29 [HTAA5]' (Does your HTA or health decision-making process include any provisions for rapidly assessing evidence, appraising evidence, and making decisions in a non-emergency context?)

Please write your answer here:

31 **Could you please describe this process further in terms of the evidence used for the appraisal and recommendation processes?**

Only answer this question if the following conditions are met:

Answer was 'Yes' at question '29 [HTAA5]' (Does your HTA or health decision-making process include any provisions for rapidly assessing evidence, appraising evidence, and making decisions in a non-emergency context?)

Please write your answer here:


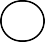


32 **Does your HTA or health decision-making process include any provisions for rapidly assessing evidence, appraising evidence, and making decisions in the case of a disaster or emergency (e.g. Covid-19 pandemic)?**

Please choose **only one** of the following:

Yes
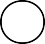
 No

33 **Can you please describe this process further?**

Only answer this question if the following conditions are met:

Answer was 'Yes' at question '32 [HTAA6]' (Does your HTA or health decision-making process include any provisions for rapidly assessing evidence, appraising evidence, and making decisions in the case of a disaster or emergency (e.g. Covid-19 pandemic)?)

Please write your answer here:


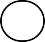


34 **Does your HTA or decision-making body include indicators to assess the impact of its own products?**

Only answer this question if the following conditions are met:

Answer was 'Yes, at the subnational level' *or* 'Yes, at the national level' at question '6 [HTAIG1]' (Does your country have a systematic formal process by which information is gathered to support healthcare decision-making?)

Please choose **only one** of the following:


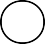
 Yes No

35 **Which of the following criteria are used by your organization to assess the impact of its recommendations?**

Only answer this question if the following conditions are met:

Answer was 'Yes' at question '34 [HTAIG13]' (Does your HTA or decision-making body include indicators to assess the impact of its own products? )

Please choose **all** that apply:

Changes within organization or facilities (training staff, buying materials, etc.) Variation in practice before and after recommendation

Variation between current practice and recommended practice over time Level of technology diffusion over time

Change in health outcomes over time (clinical changes) Changes in the cost of the medical practice over time Changes in the law

Changes in health from the point of view of the patients (perceived health, satisfaction, others)

Other:

**You can select multiple options**

# HTA Available Resources

### The section below asks about resources available for HTA and health care decision making in your country. Please answer with regards to the organization(s)/entity(ies) that you have referenced above.


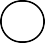


36 **Does the HTA or decision making body/bodies in your setting have an allocated budget from the public sector?**

Please choose **only one** of the following:

Yes
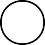
 No


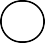
 I don't know


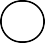


37 **From the overall budget, are any resources received through PRIVATE funding?**

Please choose **only one** of the following:

Yes


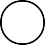
 No Private Funding

38 **What percentage of the overall budget is received through Private Funding?**

Only answer this question if the following conditions are met:

Answer was 'Yes' at question '37 [HTAIG9]' (From the overall budget, are any resources received through PRIVATE funding?)

Please write your answer(s) here: Percentage of Private Funding

39 **Out of the percentage provided above, please select the different sources of sposorship funding received by your organization from the following categories of PRIVATE institutions?**

Only answer this question if the following conditions are met:

Answer was 'Yes' at question '37 [HTAIG9]' (From the overall budget, are any resources received through PRIVATE funding?)

Please choose **all** that apply:

Industry

Research funding bodies Health care providers Academia/University Donor Agency

Medical Insurance

Other:

**If there is any funding received from a given source, please check the accompanying box.**

**You can select multiple options**

40 **How are the commissions/committees of the HTA or decision-making body funded?**

Please choose **all** that apply:

Public Budgets Application Fees

Other:


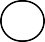


41 **Please estimate the number of professional staff involved in the HTA unit/agency/committee (in full-time equivalents).**

Please choose **only one** of the following:


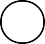
 Less than 1

1-5


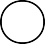
 6-20


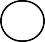
 21-50


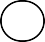
 51-100


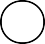
 >100

HTA Assessment (or Data)

***The following questions refer to the “Assessment” process of HTA, also linked to “Data” in the 3Ds decision framework. Assessment is defined as: A scientific process used to describe and analyse the properties of a health technology—its safety, efficacy, feasibility and indications for use, cost and***

***cost-effectiveness, as well as social, economic and ethical consequences*. *Please answer the following questions with regards to this specific process. The assessment process normally consists of evidence collection, analysis of the quality of the evidence, synthesizing the evidence, and***

***reporting the findings and implications.***


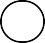


42 **How long does the assessment process take on average for any given health intervention or technology?**

Please choose **only one** of the following:

<1 month


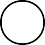
 1-3 months


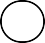
 3-6 month


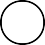
 6-12 months


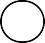
 >1 year

43 **In the last 12 months, approximately, how many assessments were performed?**

Please write your answer here:

**es**

44 **Of the number provided above, please provide the total number of assessments performed in the last 12 months under each of the following categories:**

**To the best degree possible, the total should match the answer provided in previous question.**

**Population Level Health Interventions(i.e. large scale screening/vaccination programs)**

|  | **Number of Assessments** |
| --- | --- |
| **Pharmaceuticals/Medicin** |  |
| **Medical Procedure** |  |
| **Medical devices** |  |
| **Diagnostic Tests** |  |
| **Population Level Health Interventions** |  |
| **Others (Please Specify)** |  |

45 Other HTAIG18a:

Please write your answer here:

# HTA Appraisal (or Dialogue)

***The following questions refer to the “Appraisal” process of HTA, also linked to “Dialogue” in the 3Ds decision framework. In the Appraisal phase, the evidence that is provided from the previous assessment is scrutinized and deliberated on. Please answer the following questions with regards to this specific process. In the appraisal step, the HTA organization interprets the results of the assessment and formulates a recommendation or guidance to inform decision makers.***

46 Please list which of the following aspects are covered in the appraisal phase for the categories of interventions?

|  | **Pharmace Medicines** | **Medical Procedur** | **Medical Devices** | **Diagnostic Tests** | **Population Level**  **Health Intervention** |
| --- | --- | --- | --- | --- | --- |
| **Safety** |  |  |  |  |  |
| **Clinical effectiveness** |  |  |  |  |  |
| **Cost-Effectiveness Analysis (Economic Evaluations)** |  |  |  |  |  |
| **Budget Impact Analysis** |  |  |  |  |  |
| **Equity issues (e.g. Socioeconomic considerations, Gender)** |  |  |  |  |  |
| **Severity of Illness** |  |  |  |  |  |
| **Financial Risk Protection** |  |  |  |  |  |
| **Other Ethical issues** |  |  |  |  |  |
| **Feasibility (e.g. availability of budget and human resources)** |  |  |  |  |  |
| **Acceptability to providers** |  |  |  |  |  |
| **Acceptability to patients** |  |  |  |  |  |
| **Others (Please Specify)** |  |  |  |  |  |

### s

**uticals/**

**es**

*Population Level Health Interventions - i.e. large scale screening/vaccination programs*

47 Other 1

Please write your answer here:


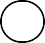


48 **Are there national guidelines for the preparation of economic evaluations?**

Please choose **only one** of the following:


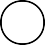
 Yes, Please provide a link in the box or upload a file in the next question No

Make a comment on your choice here:

**If yes, please provide a link in the box or upload a file in the next question (an upload button will appear once you click yes here)**

49 If necessary, please upload a file of national guidelines for the preparation of economic evaluations

Only answer this question if the following conditions are met:

Answer was 'Yes, Please provide a link in the box or upload a file in the next question' at question '48 [HTAA1a]' (Are there national guidelines for the preparation of economic evaluations? )

Kindly attach the aforementioned documents along with the survey


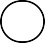


50 **Are there other relevant guidelines for the preparation of evidence for the appraisal process?**

Please choose **only one** of the following:

Yes, Please provide a link in the box or upload a file in the next question
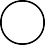
 No

Make a comment on your choice here:

**If yes, please provide a link in the box or upload a file in the next question (an upload button will appear once you click yes here)**

51 If necessary, please upload a file of other relevant guidelines for the preparation of evidence

Only answer this question if the following conditions are met:

Answer was 'Yes, Please provide a link in the box or upload a file in the next question' at question '50 [HTAA1]' (Are there other relevant guidelines for the preparation of evidence for the appraisal process? )

Kindly attach the aforementioned documents along with the survey


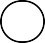


52 **Does your country have a public and updated database of health technology costs or prices?**

Please choose **only one** of the following:

Yes, Please provide a link in the box or upload a file in the next question
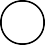
 No

Make a comment on your choice here:

**If yes, please provide a link in the box or upload a file in the next question (an upload button will appear once you click yes here)**

53 If necessary, please upload a file of your database of health technology costs or prices

Only answer this question if the following conditions are met:

Answer was 'Yes, Please provide a link in the box or upload a file in the next question' at question '52 [HTAIG11]' (Does your country have a public and updated database of health technology costs or prices?)

Kindly attach the aforementioned documents along with the survey


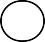


54

**If cost-effectiveness or cost-utility analysis is used for decision-making (i.e. if an incremental cost effectiveness ratio is computed), is there an officially endorsed threshold used to assess whether a new procedure, a new medicine, or a new device will be funded?**

Please choose **only one** of the following:

Yes
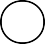
 No


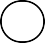


55

**Does the threshold vary across different categories of patients, diseases or interventions?**

Only answer this question if the following conditions are met:

Answer was 'Yes' at question '54 [HTAR7]' ( If cost-effectiveness or cost-utility analysis is used for decision-making (i.e. if an incremental cost effectiveness ratio is computed), is there an officially endorsed threshold used to assess whether a new procedure, a new medicine, or a new device will be funded? )

Please choose **only one** of the following:

Yes
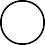
 No

56 **What is the basis for the variation?**

Only answer this question if the following conditions are met:

Answer was 'Yes' at question '55 [HTAR7a]' ( Does the threshold vary across different categories of patients, diseases or interventions? )

Please write your answer here:

57

**Please specify the cost-effectiveness threshold(s) used. Also, please specify since when the threshold(s) have been in use and how often are they reviewed.**

Only answer this question if the following conditions are met:

Answer was 'Yes' at question '54 [HTAR7]' ( If cost-effectiveness or cost-utility analysis is used for decision-making (i.e. if an incremental cost effectiveness ratio is computed), is there an officially endorsed threshold used to assess whether a new procedure, a new medicine, or a new device will be funded? )

Please write your answer here:

58 **Please explain the rationale for using this threshold value in your country?**

Only answer this question if the following conditions are met:

Answer was 'Yes' at question '54 [HTAR7]' ( If cost-effectiveness or cost-utility analysis is used for decision-making (i.e. if an incremental cost effectiveness ratio is computed), is there an officially endorsed threshold used to assess whether a new procedure, a new medicine, or a new device will be funded? )

Please write your answer here:

**You can also provide a link to a document explaining the rationale in the comment box or upload the relevant document in the following question**

**es**

60 **Please fill in the following table regarding the appraisal organization for each category of health intervention.**

**Kindly refer back to the organizations that you mentioned in question 19 of the Institutions and**

**Governance section.**

59 If necessary, please upload a file of explaining the rationale for using specific values for cost-effectiveness.

Only answer this question if the following conditions are met:

Answer was 'Yes' at question '54 [HTAR7]' ( If cost-effectiveness or cost-utility analysis is used for decision-making (i.e. if an incremental cost effectiveness ratio is computed), is there an officially endorsed threshold used to assess whether a new procedure, a new medicine, or a new device will be funded? )

Kindly attach the aforementioned documents along with the survey

|  | **Who appoints the members of each appraisal**  **commission/committee?** | **How often does each appraisal commission/committee meet?** |
| --- | --- | --- |
| **Pharmaceuticals/Medicin** |  |  |
| **Medical Procedures** |  |  |
| **Medical Devices** |  |  |
| **Diagnostic Tests** |  |  |
| **Population Level Health Interventions(i.e. large scale screening/vaccination programs)** |  |  |
| **Others(Please Specify)** |  |  |

61 Other

Please write your answer here:

62 Which of the stakeholders below (see table) are represented in the body (Commission, Committee, etc.) responsible for the appraisal of applications?

**uticals**

|  | **Pharmace**  **/ Medicine** | **Medical Procedure** | **Medical Device** | **Diagnostic Tests** | **Population Level**  **Health Intervention** |
| --- | --- | --- | --- | --- | --- |
| **Medical professionals (doctors, pharmacists, dentists…)** |  |  |  |  |  |
| **Public health specialists, epidemiologists** |  |  |  |  |  |
| **Statisticians** |  |  |  |  |  |
| **Economists** |  |  |  |  |  |
| **Specialists in social science (ethics, philosophy, etc.)** |  |  |  |  |  |
| **Government** |  |  |  |  |  |
| **Social health insurance** |  |  |  |  |  |
| **Private insurers** |  |  |  |  |  |
| **Medical association/chamber** |  |  |  |  |  |
| **Medical Union** |  |  |  |  |  |
| **Hospital association** |  |  |  |  |  |
| **Pharma/medical industry** |  |  |  |  |  |
| **Patient association** |  |  |  |  |  |
| **Consumer association** |  |  |  |  |  |
| **Representative of citizens** |  |  |  |  |  |

### s

**uticals**

**s**

*Population Level Health Interventions - i.e. large scale screening/vaccination programs*

|  | **Pharmace**  **/ Medicine** | **Medical Procedure** | **Medical Device** | **Diagnostic Tests** | **Population Level**  **Health Intervention** |
| --- | --- | --- | --- | --- | --- |
| **Vulnerable and**  **marginalized groups (e.g. religious**  **minorities, migrants, rural populations, women’s’ groups, youth groups etc)** |  |  |  |  |  |
| **Other members** |  |  |  |  |  |

63 Other

Please write your answer here:


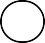


64 **Do all the stakeholders represented in these bodies have equal voice in the appraisal process (e.g. by vote, allocated time, or other mode of input)?**

Please choose **only one** of the following:


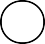
 Yes No

65 Which of the following are the stakeholders who have less voice in the appraisal process (e.g. by less voting power, allocated time, or other mode of input)?

##### Only answer this question if the following conditions are met:

Answer was 'No' at question '64 [HTAAP1c]' (Do all the stakeholders represented in these bodies have equal voice in the appraisal process (e.g. by vote, allocated time, or other mode of input)? )

Please choose the appropriate response for each item:

|  | **Have less voice** |
| --- | --- |
| **Medical professionals (doctors, pharmacists, dentists…)** | 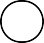 |
| **Public health specialists, epidemiologists** | 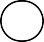 |
| **Statisticians** | 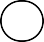 |
| **Economists** | 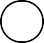 |
| **Specialists in social science (ethics, philosophy, etc.)** | 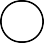 |
| **Government** | 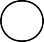 |
| **Social health insurance** | 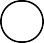 |
| **Private insurers** | 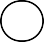 |
| **Medical association/chamber** | 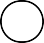 |
| **Medical Union** | 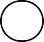 |
| **Hospital association** | 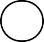 |
| **Pharma/medical industry** | 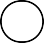 |
| **Patient association** | 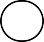 |
| **Consumer association** | 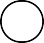 |
| **Representative of citizens** | 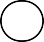 |

|  | **Have less voice** |
| --- | --- |
| **Vulnerable and**  **marginalized groups (e.g. religious**  **minorities, migrants, rural populations, women’s’ groups, youth groups etc)** | 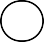 |
| **Other members:** | 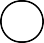 |


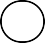


66 **Are stakeholders not represented in the body responsible for the appraisal of applications invited to react/comment during the whole process?**

Please choose **only one** of the following:

Yes
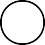
 No

67

**Referring to the question above, How is this done?**

Only answer this question if the following conditions are met:

Answer was 'Yes' at question '66 [HTAAP1e]' (Are stakeholders not represented in the body responsible for the appraisal of applications invited to react/comment during the whole process? )

Please choose **all** that apply:

Through public consultations Through specific hearings Through online submissions

Other:

**Please specify if "Other" in the accompanying box**


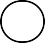


68 **Are members of the appraisal body required to provide a declaration of conflict of interest?**

Please choose **only one** of the following:

Yes
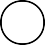
 No

HTA Recommendation (or Decision)

***The following questions refer to the “Recommendation” process of HTA, also linked to “Decision” in the 3Ds decision framework. In this phase, recommendations are developed based on the appraisal. Please answer the following questions with regards to this specific phase.***


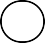


69 **Is there a separate committee or entity responsible for recommendation (or decision) after the appraisal process has been conducted?** *

Please choose **only one** of the following:

Yes
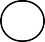
 No

**It is possible for the appraisal committee to have a decision role as well - If that is the case in your setting, please select No as we have already asked questions about this entity in the previous section.**

70

Please fill in the following table regarding the recommendation process for each category of health intervention.

##### Only answer this question if the following conditions are met:

Answer was 'Yes' at question '69 [HTAAP1]' (Is there a separate committee or entity responsible for recommendation (or decision) after the appraisal process has been conducted?)

|  | **Who appoints the members of each commission/committee involved in the recommendation process?** |
| --- | --- |
| **Pharmaceuticals / Medicines** |  |
| **Medical Procedures** |  |
| **Medical Devices** |  |
| **Diagnostic Tests** |  |
| **Population Level Health Interventions (i.e. large-scale screening/vaccination programs)** |  |
| **Others (Please Specify)** |  |

Kindly refer back to the organization that you mentioned in question 19 of the Institutions and Governance section.

71 OtherHTAR1aa

Please write your answer here:

72 Which of the stakeholders below are represented in the body (Commission, Committee, etc.) which provides the final decision?

##### Only answer this question if the following conditions are met:

Answer was 'Yes' at question '69 [HTAAP1]' (Is there a separate committee or entity responsible for recommendation (or decision) after the appraisal process has been conducted?)

|  | **Pharmace Medicine** | **Medical Procedur** | **Medical Device** | **Diagnostic Tests** | **Population Level**  **Health Intervention** |
| --- | --- | --- | --- | --- | --- |
| **Medical professionals (doctors, pharmacists, dentists…)** |  |  |  |  |  |
| **Public health specialists, epidemiologists** |  |  |  |  |  |
| **Statisticians** |  |  |  |  |  |
| **Economists** |  |  |  |  |  |
| **Specialists in social science (ethics, philosophy, etc.)** |  |  |  |  |  |
| **Government** |  |  |  |  |  |
| **Social health insurance** |  |  |  |  |  |
| **Private insurers** |  |  |  |  |  |
| **Medical association/chamber** |  |  |  |  |  |
| **Medical Union** |  |  |  |  |  |
| **Hospital association** |  |  |  |  |  |
| **Pharma/medical industry** |  |  |  |  |  |
| **Patient association** |  |  |  |  |  |
| **Consumer association** |  |  |  |  |  |
| **Representative of citizens** |  |  |  |  |  |

**s**

**uticals/**

**es**

**uticals/**

**es**

**s**

*Population Level Health Interventions - i.e. large-scale screening/vaccination programs*

|  | **Pharmace Medicine** | **Medical Procedur** | **Medical Device** | **Diagnostic Tests** | **Population Level**  **Health Intervention** |
| --- | --- | --- | --- | --- | --- |
| **Vulnerable and**  **marginalized groups (e.g. religious**  **minorities, migrants, rural populations, women’s’ groups, youth groups etc)** |  |  |  |  |  |
| **Other members** |  |  |  |  |  |

73 OtherHTAR1b

Please write your answer here:


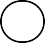


74 **Are members of the recommendation body required to provide a declaration of conflict of interest?**

Only answer this question if the following conditions are met:

Answer was 'Yes' at question '69 [HTAAP1]' (Is there a separate committee or entity responsible for recommendation (or decision) after the appraisal process has been conducted?)

Please choose **only one** of the following:


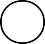
 Yes No


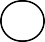


75 **Do all the stakeholders represented in these bodies have equal voice in the process (e.g. by vote, allocated time, or other mode of input)?**

Only answer this question if the following conditions are met:

Answer was 'Yes' at question '69 [HTAAP1]' (Is there a separate committee or entity responsible for recommendation (or decision) after the appraisal process has been conducted?)

Please choose **only one** of the following:

Yes
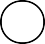
 No

76

Which of the following are the stakeholders who have less voice in this process (e.g. by less voting power, allocated time, or other mode of input)?

##### Only answer this question if the following conditions are met:

Answer was 'No' at question '75 [HTAR1c]' (Do all the stakeholders represented in these bodies have equal voice in the process (e.g. by vote, allocated time, or other mode of input)? )

Please choose the appropriate response for each item:

|  | **Have less voice** |
| --- | --- |
| **Medical professionals (doctors, pharmacists, dentists…)** | 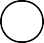 |
| **Public health specialists, epidemiologists** | 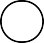 |
| **Statisticians** | 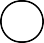 |
| **Economists** | 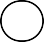 |
| **Specialists in social science (ethics, philosophy, etc.)** | 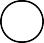 |
| **Government** | 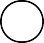 |
| **Social health insurance** | 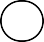 |
| **Private insurers** | 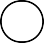 |
| **Medical association/chamber** | 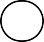 |
| **Medical Union** | 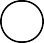 |
| **Hospital association** |  |
| **Pharma/medical industry** |  |
| **Patient association** |  |
| **Consumer association** |  |
| **Representative of citizens** |  |

|  | **Have less voice** |
| --- | --- |
| **Vulnerable and**  **marginalized groups (e.g. religious**  **minorities, migrants, rural populations, women’s’ groups, youth groups etc)** |  |
| **Other members:** |  |

77 **Is there a possibility to appeal against the decision?**

Only answer this question if the following conditions are met:

Answer was 'Yes' at question '69 [HTAAP1]' (Is there a separate committee or entity responsible for recommendation (or decision) after the appraisal process has been conducted?)

Please choose **only one** of the following:

Yes No

78 **Before which body can the decision be challenged?**

Only answer this question if the following conditions are met:

Answer was 'Yes' at question '77 [HTAR4]' (Is there a possibility to appeal against the decision? )

Please choose **all** that apply:

HTA Body Judicial Court

Other:

**Please specify if "Other" in the accompanying box**

79 **Are the following published and readily available? Please provide links with your answers**

Please choose all that apply and provide a comment: Minutes of the meetings

Assessment reports

Recommendations (or decisions where relevant)

Rationale for the decision

Other, Please specify

No outputs from the recommendation process are published

**Please provide links in the box**

You can select multiple options

Barriers to Health Technology Assessment

80

**Please select and rank, from the list below, which barriers are most prominent at the country level to the *use of* HTA (or related decision-making processes) in health care policy decision- making. The lack of:**

Please number each box in order of preference from 1 to 6

Awareness/Advocacy of the importance of HTA Institutionalization of HTA

Mandate from Policy Authority Political support

Qualified human resources

Other Issues

**Please drag or double click the options in order of priority, with the highest priority being on the top.**

81

**Please select and rank in order of priority, from the list below, which barriers are most prominent at the level of the HTA (or related decision-making processes) organization in terms**

***of production capabilities.* The lack of:**

Please number each box in order of preference from 1 to 5

Budget availability Data Availability Knowledge of methods

Dedicated human resources

Other Issues

**Please drag or double click the options in order of priority, with the highest priority being on the top.**

82

**Which of the following academic or training programs in your country to support HTA and health decision-making require further development? Please rank in order of priority:**

Please number each box in order of preference from 1 to 4

Courses/seminars/workshops Higher education/Masters

Internal staff training sessions or workshops

Other

**Please drag or double click the options in order of priority, with the highest priority being on the top.**

83 **Which of the following areas would benefit most from HTA capacity building activities from WHO or other organizations?**

Please choose **all** that apply:

Mandate Development Legal Framework Institutionalization Assessment Methodology Appraisal Process Stakeholder Engagement

Monitoring & Evaluation Process

Economic Evaluation & Budget Impact Analysis

Other:

84 **If you would like to mention any further barriers related to HTA in your setting, please describe them here.**

Please write your answer here:

**If you would like to specify any of the "Other Issues" that may have been ranked above, please do so here.**

Final Comments and Authorization

Please add any general comments on the survey:

Please write your answer here:

**Please tick:**

*

Please choose **all** that apply:

We consider the information to be true to the best of our knowledge We authorize WHO to use any information submitted in this survey

I confirm that I am submitting these responses as the nominated focal point respondent, OR that I have authority delegated from the nominated focal point respondent to provide responses to the questions in this survey.

**CHECK ALL BOXES**

**WHO HQ would like to conduct deep dives with some country respondents to further enrich our understanding of the HTA**

**process. Would you be willing to participate and assist us in this process? Please respond:**

*

Please choose **only one** of the following:

Yes No

**Thank you for your valuable time and participation in this survey.**

Submit your survey.

Thank you for completing this survey.
