# Supplementary material for: The global landscape of country-level health technology assessment processes: A survey among 104 countries
Source: Health Policy Open. 2025 Mar 27;8:100138. doi: 10.1016/j.hpopen.2025.100138 (PMC11999493; doi:10.1016/j.hpopen.2025.100138)
Supplement: Supplementary Data 7 [file mmc7.docx]

Figure 3: Top ranked barrier (first three) for HTA use (top) and production (bottom).


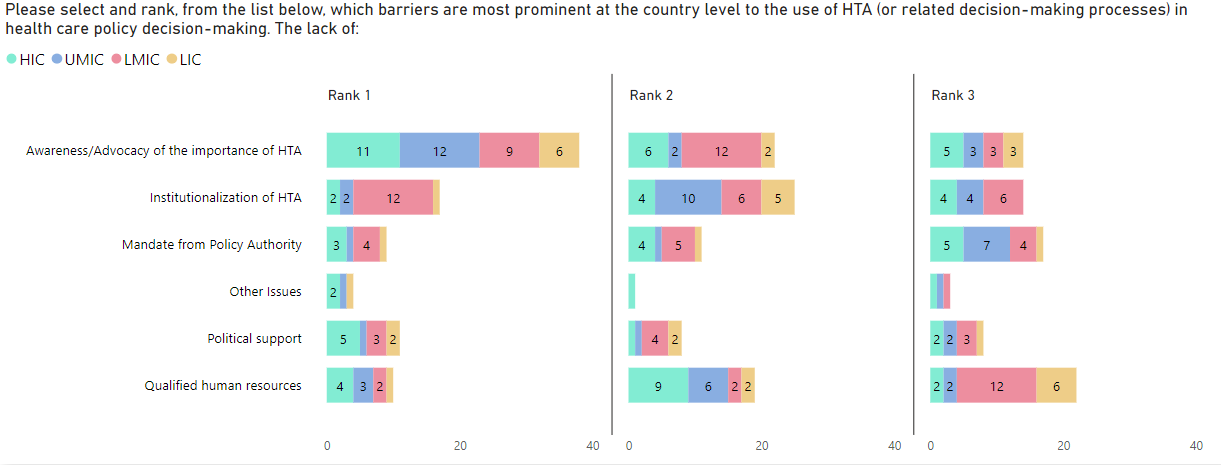


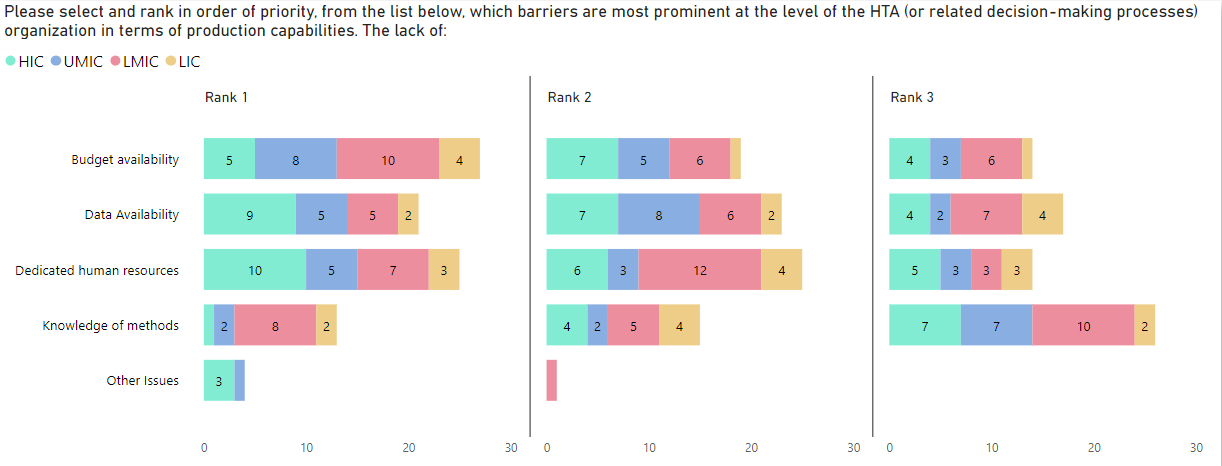


Caption: Only top-ranked barriers are included in this figure. For full rankings of barriers please refer to the survey database.
